# SsPit2A/B Effectors from *Sporisorium scitamineum* Interact with the Sugarcane PLCP ScRD21A and Reduce ScRD21A-Associated Cysteine Protease Activity via a Conserved LXRR Motif

**DOI:** 10.3390/plants15091408

**Published:** 2026-05-05

**Authors:** Yangmin Zhu, Zengrong Huang, Junyi Wen, Jiangming Wei, Ke Liu, Yuan Su, Yunfeng Liu, Shengchao Ge

**Affiliations:** State Key Laboratory for Conservation and Utilization of Subtropical Agro-Bioresources, College of Life Science and Technology, Guangxi University, Nanning 530004, China; drow1219@163.com (Y.Z.); 2408301029@st.gxu.edu.cn (Z.H.); 2308301076@st.gxu.edu.cn (J.W.); weijm1949@163.com (J.W.); 13170148566@163.com (K.L.); suyuan@gxu.edu.cn (Y.S.)

**Keywords:** *Saccharum spontaneum*, *Sporisorium scitamineum*, Pit2, RD21, PLCP, plant immunity

## Abstract

Papain-like cysteine proteases (PLCPs) are central immune hubs frequently targeted by pathogen effectors. Sugarcane smut, caused by *Sporisorium scitamineum*, threatens global sugarcane yield, yet effector manipulation of host PLCPs remains unclear. Genome-wide analysis of *Saccharum spontaneum* AP85-441 identified 61 PLCP-encoding genes, which were classified into nine conserved subfamilies. Among these, ScRD21A, a member of the RD21 subfamily, was prioritized for functional characterization. Two Pit2 homologs, SsPit2A and SsPit2B, were identified from *S. scitamineum*. Yeast two-hybrid, BiFC and pull-down assays demonstrated that both effectors interact with ScRD21A, and that this interaction depends on a conserved LXRR motif within their PID14-like region. In total protein extracts from *Nicotiana benthamiana*, co-expression of SsPit2A or SsPit2B reduced ScRD21A-associated cysteine protease activity. Transient expression of ScRD21A enhanced flg22-induced ROS production, attenuated *Pst DC3000*-induced hypersensitive response-associated necrosis, and increased resistance to *Botrytis cinerea*. Together, these results support a conserved PLCP-targeting strategy in smut fungi and identify the ScRD21A–SsPit2A/B module as a tractable framework for studying effector–protease interactions relevant to sugarcane smut.

## 1. Introduction

Sugarcane (*Saccharum* spp. hybrids) is one of the most economically important C4 crops worldwide, supplying more than 80% of global sugar production and serving as a critical biomass feedstock for bioenergy and bioproducts [1,2]. Cultivated on over 26 million hectares across tropical and subtropical regions, sugarcane supports the livelihoods of millions of smallholder farmers, particularly in major producing countries such as Brazil, India, China, and Thailand [2]. As sessile organisms, plants—including sugarcane—are continuously exposed to a wide array of microbial pathogens, with initial host–pathogen interactions often occurring at epidermal surfaces and within the apoplast [3]. Among the major biotic stresses limiting sugarcane productivity, sugarcane smut, caused by the obligate biotrophic basidiomycete fungus *S. scitamineum*, is particularly destructive. The pathogen preferentially infects young buds, establishes systemic colonization through vascular tissues, and ultimately induces the formation of a characteristic whip-like sorus filled with teliospores. Under warm and humid environmental conditions, smut epidemics can result in yield losses ranging from 10% to 50%, with severe outbreaks occasionally causing complete crop failure in susceptible cultivars [4,5]. Beyond direct yield reduction, smut infection also decreases sucrose content and compromises ratoon crop performance, imposing substantial economic and agronomic burdens on the global sugar industry [2,4,5].

Plants have evolved a sophisticated two-layered innate immune system, consisting of pattern-triggered immunity (PTI) and effector-triggered immunity (ETI) [6]. PTI is initiated at the plant plasma membrane by pattern recognition receptors (PRRs), which recognize conserved pathogen-associated molecular patterns (PAMPs), such as flg22 (a conserved 22-amino-acid epitope derived from bacterial flagellin) or fungal chitin [6]. A hallmark early PTI response is the rapid production of reactive oxygen species (ROS) by the NADPH oxidase RESPIRATORY BURST OXIDASE HOMOLOG D (RBOHD), which reinforces cell walls, promotes stomatal closure, and activates downstream defense signaling [7,8]. However, host-adapted pathogens deliver secreted effector proteins into host cells to suppress PTI. For example, the effector AvrPtoB directly targets PRRs to disrupt immune signaling cascades [9]. To counteract effector-mediated immune suppression, plants activate ETI via intracellular nucleotide-binding domain and leucine-rich repeat (NLR) receptors, which directly or indirectly recognize cognate effectors [10]. This process typically triggers localized programmed cell death (PCD), the hypersensitive response (HR), and subsequent systemic acquired resistance (SAR) [11]. Accumulating evidence has demonstrated that PTI and ETI function synergistically rather than as independent, uncoupled pathways [8]. RBOHD-dependent apoplastic ROS burst acts as a core signaling node that connects these two immune branches and fine-tunes the amplitude and duration of plant immune responses [12]. This dynamic immune interplay is continuously shaped by the adaptive evolution of pathogens, driving the perpetual molecular arms race between host plants and their co-evolving pathogens [13].

Within this immune network, papain-like cysteine proteases (PLCPs, MEROPS family C1A) also participate in plant growth, development, and stress adaptation, while serving important functions in antimicrobial defense, immune signal amplification, and stress-associated programmed cell death [14,15]. PLCPs are synthesized as inactive preproproteins containing an N-terminal signal peptide, an autoinhibitory prodomain, and a mature catalytic domain [16]. In land plants, they are phylogenetically grouped into nine major subfamilies (AALP, CEP, CTB, RD19, RD21, SAG12, THI, XBCP, and XCP), each exhibiting distinct subcellular localization, substrate specificity, and physiological roles [17,18]. Among them, RD21-like PLCPs are particularly notable because they repeatedly emerge as pathogen effector targets in diverse pathosystems [14].

Recent studies have strengthened the view that RD21 proteins are immune-relevant proteases rather than merely general degradative enzymes. In Arabidopsis, RD21 contributes positively to resistance against bacterial and fungal pathogens [15]. In wheat and Nicotiana systems, RD21-related proteolysis has been linked to the release of defense-active peptides and to amplification of ROS-associated antiviral defense [19,20]. RD21 abundance and activity are also regulated by post-translational mechanisms, including ubiquitination-dependent turnover by SINAT4 and stabilization by the deubiquitinase OTU2 [21,22]. Conversely, pathogens can directly target RD21-type proteases; for example, the root-knot nematode effector MiCE108 promotes degradation of Arabidopsis RD21A to suppress host defense [23]. These findings collectively position RD21-like proteases at an important interface between host immunity and pathogen virulence [15].

Time-ordered gene co-expression network analysis following *S. scitamineum* infection revealed higher transcript abundance of *PLCPs* in the smut-resistant sugarcane cultivar YT93-159 than in the susceptible cultivar ROC22, particularly at late infection stages [24]. Research on sugarcane proteases remains relatively limited, with previous studies focusing mainly on protease inhibitors such as cystatins [25]. Proteomic analyses have identified multiple proteases in sugarcane stem cell walls [26]. To our knowledge, genome-wide identification and systematic functional characterization of proteases, particularly the PLCP family, remain scarce in sugarcane, likely due to its extreme polyploidy and genome complexity [1,27]. Similarly, functional characterization of *S. scitamineum* effectors has also been confined to only a few candidates, and many reported effectors remain at the stage of genome-based prediction [28,29].

The genome of *S. scitamineum* shares close genomic homology with that of *Ustilago maydis* (maize smut fungus), and homologs of effectors such as Pep1, Cmu1, and Tin2 have been identified with high sequence similarity [29]. These effectors play pivotal virulence roles during smut infection: SsPEP1 inhibits the activity of sugarcane POD-1a to alleviate ROS burst, interferes with mating and mitogen-activated protein kinase (MAPK) signaling pathways, and promotes early colonization [30]. Moreover, these effectors show varied subcellular localization patterns, targeting distinct host compartments or structures such as the nucleoplasm, mitochondria, and cell wall. For instance, g5159 localizes to the cell wall and inhibits AvrB-induced ETI, while g3890 interacts with host protein phosphatase 2A (PP2A) subunits and endochitinases to disrupt immune signaling [28]. Evolutionary analysis has shown that genes such as *Srt1* and *Pit1* are driven by either positive selection or intermittent selection, evading host recognition through polymorphism [31]. Recently, it has also been found that SsPE15 hijacks ScVSR1-mediated vacuolar vesicular trafficking to escape immune surveillance [32]; SsEF83, a cysteine-rich effector, induces cell death in *N. benthamiana*, and its deletion significantly impairs virulence [33].

Given the crucial role of PLCPs as central immune hubs targeted by pathogen effectors in diverse plant–pathogen interactions, and the lack of systematic research on sugarcane PLCPs and their regulation by *S. scitamineum* effectors, this aspect remains poorly understood in sugarcane. Building on the genomes of *S. spontaneum* AP85-441, the present study aimed to systematically identify the sugarcane PLCP gene family, characterize the functional role of key PLCP members, and explore the interaction between *S. scitamineum* effectors and sugarcane PLCPs. This study is expected to improve our understanding of effector–PLCP interactions in the sugarcane–*S. scitamineum* pathosystem and to highlight candidate molecular components potentially relevant to sugarcane smut resistance improvement.

## 2. Results

### 2.1. Genome-Wide Identification, Phylogenetic Classification, and Evolutionary Expansion Mechanisms of the ScPLCP Family

**Figure 1:**
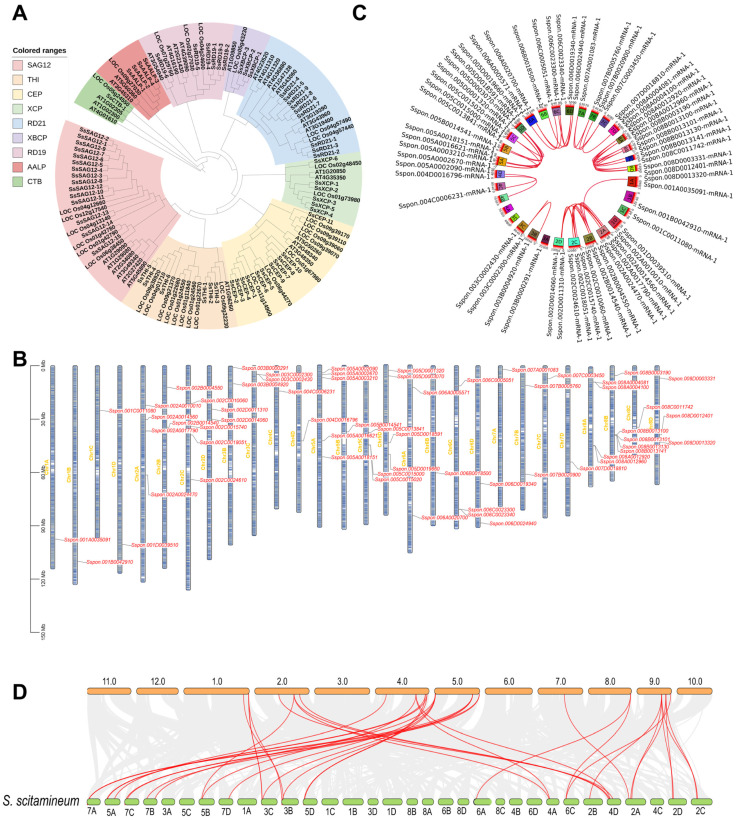
Identification and family analyses of sugarcane PLCPs. (**A**) Maximum likelihood phylogenetic tree of PLCP proteins from sugarcane (*S. spontaneum* AP85-441), *Arabidopsis thaliana* (ecotype Columbia-0) and *Oryza sativa* ssp. *japonica* cv. Nipponbare. Subfamily-specific clades are color-coded. (**B**) Chromosomal distribution of the 61 ScPLCP genes across the 28 chromosomes of sugarcane. Genes are arranged vertically by chromosomal position. The scale bar on the left indicates chromosome length (Mb). (**C**) Synteny analysis of ScPLCP genes within the sugarcane genome. Red lines highlight synteny specifically among ScPLCP genes. (**D**) Synteny analysis between the sugarcane genome and the monocot model plant rice. Gray background lines represent all syntenic relationships between the two species, while red lines indicate synteny between PLCP genes of sugarcane and rice.

Using HMMER searches for the Peptidase_C1 domain followed by reciprocal BLASTP validation, we identified 61 non-redundant *PLCP* genes in the monoploid *S. spontaneum* AP85-441 genome (Table 1). A maximum likelihood (ML) phylogenetic tree constructed with Arabidopsis and rice PLCPs separated the sugarcane proteins into nine subfamilies: AALP, CEP, CTB, RD19, RD21, SAG12, THI, XBCP, and XCP (Figure 1A). The major subfamily groupings were consistent with those obtained from the neighbor-joining (NJ) analysis (Figure S1), whereas some topological differences were observed at deeper phylogenetic levels (e.g., the phylogenetic position of XBCP). Proteins within the same subfamily exhibit high similarity (Figure S2). All nine canonical PLCP lineages represented in Arabidopsis and rice were also present in sugarcane, indicating overall conservation of major family structure across these species [18,34].

Physicochemical analysis showed that most ScPLCP proteins are 300–400 aa in length and 37–50 kDa in predicted molecular weight. Extreme variations were observed in specific members, such as RD21-6 (1533 aa, 165.4 kDa) and RD19-4 (177 aa) (Table 1), suggesting specialized evolutionary adaptations through domain fusion or truncation [18]. The predicted isoelectric points varied across subfamilies, with SAG12 members generally acidic (pI 4.69–6.03) and AALP members spanning a broader range (pI 5.59–8.3) (Table 1). Taken together, these results demonstrate that the sugarcane PLCP family exhibits detectable divergence in protein size and charge properties, and this charge variation suggests that distinct ScPLCP subfamilies may be adapted to exert their biological functions efficiently in disparate subcellular compartments (e.g., the acidic vacuole).

Chromosomal mapping showed that the 61 *ScPLCP* genes are distributed unevenly across the 28 chromosomes of *S. spontaneum*, with most chromosomes carrying 1–3 members and chromosome 8B containing five loci (Figure 1B). Given the highly autopolyploid nature of the sugarcane genome, we further investigated the mechanisms underlying *ScPLCP* family expansion. Intra-genomic synteny analysis identified 42 collinear gene pairs, supporting the conclusion that segmental duplication and whole-genome duplication, rather than tandem duplication, were the predominant contributors to the expansion of the *ScPLCP* family (Figure 1C). This pattern agrees with the extensive polyploidization history of the *Saccharum* lineage [1]. Interspecific synteny analysis identified 34 orthologous gene pairs between sugarcane and rice, supporting substantial conservation of PLCP-associated syntenic blocks between the two genomes (Figure 1D).

### 2.2. Structural Features and Promoter Analysis of the ScPLCP Family

**Figure 2:**
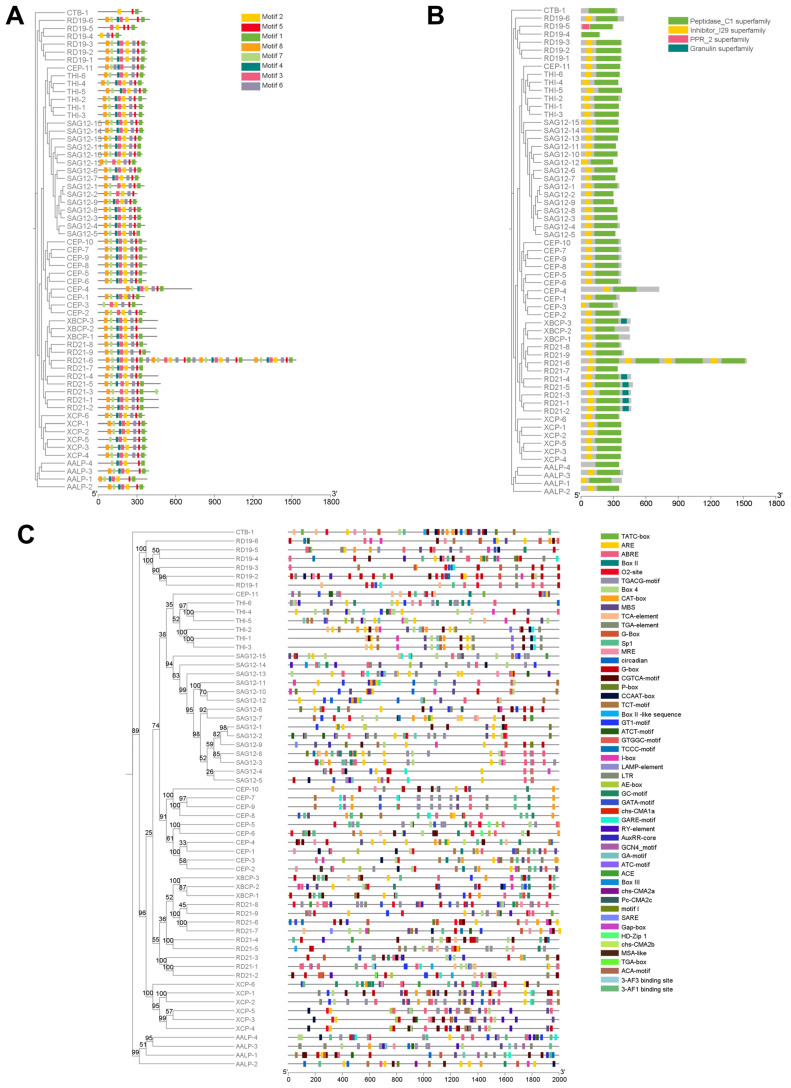
Conserved structural architecture and transcriptional regulatory landscape of ScPLCPs. (**A**) Phylogenetic tree and conserved motif analysis of ScPLCP family. Different colors indicate distinct motifs. Scale bar represents protein length (amino acids). (**B**) Phylogenetic tree and conserved domain analysis of ScPLCP family. Different colors denote different conserved domains. Scale bar indicates protein length. (**C**) Cis-regulatory element distribution in *ScPLCP* promoter regions. Colored boxes represent hormone- and stress-related cis-elements. Scale bar shows promoter length.

All identified ScPLCPs contained the characteristic Peptidase_C1 (PF00112) catalytic domain, and most of them possessed a detectable N-terminal Inhibitor_I29 (PF08246) pro-domain (Figure 2B). MEME analysis identified eight conserved motifs; motifs 1–6 mapped mainly to the catalytic region, whereas motifs 7 and 8 corresponded mainly to the pro-peptide autoinhibitory region (Figure 2A,B, Supplementary Table S3). In addition, certain members harbor extra domains, including a C-terminal Granulin (GRAN) domain in XCP-3 and several RD21 proteins, as well as a pentatricopeptide repeat (PPR) domain in RD19-5, indicating structural diversification within the family [18].

Promoter analysis of the 2000 bp upstream regions revealed that *ScPLCP* genes are enriched in hormone- and stress-responsive cis-acting regulatory elements (Figure 2C). Abscisic acid (ABA)-responsive elements were present in 91.8% of promoters, and methyl jasmonate (MeJA)-responsive motifs were present in 83.6%. Light-responsive elements (G-box, Box 4) and abiotic stress-related motifs, such as anaerobic-responsive elements (83.6%) and drought-responsive MBS elements (73.8%), were also common. Collectively, these findings suggest that transcription of *ScPLCP* genes is likely regulated by multiple hormonal and environmental cues.

### 2.3. ScRD21A Was Prioritized as a Representative Sugarcane RD21-like Candidate for Downstream Characterization

**Figure 3:**
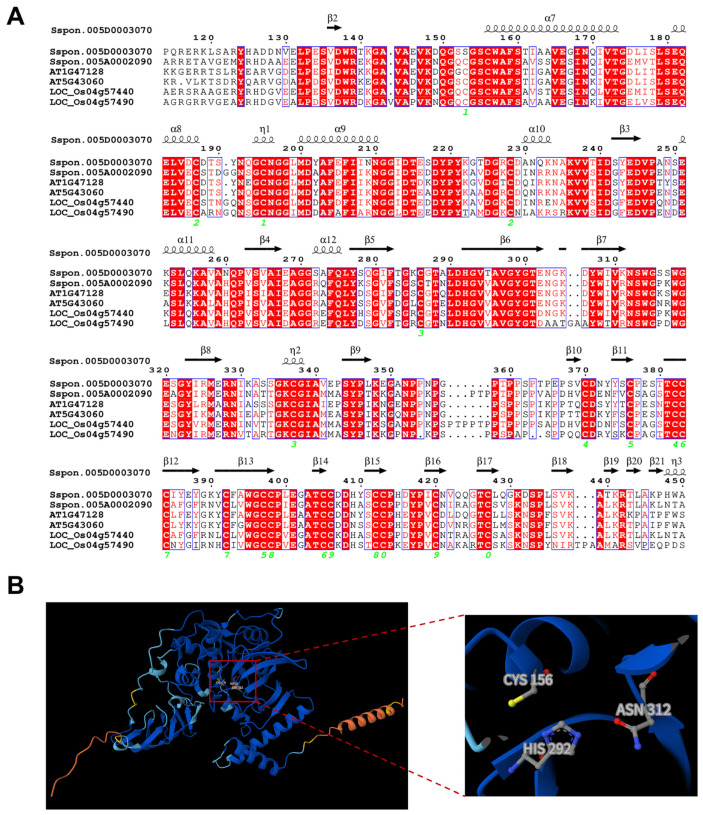
Structural characterization of ScRD21A. (**A**) Multiple sequence alignment of RD21 subfamily members from sugarcane, rice, and Arabidopsis generated with MEGA and visualized using ESPript 3.0. Strictly conserved residues are shaded red. Secondary structure elements are shown above the alignment based on the predicted three-dimensional structure of sugarcane RD21 member Sspon.005D0003070. Disulfide bridges are indicated by green numbers below the sequences, with identical numbers denoting paired cysteine residues. (**B**) AlphaFold3 predicted three-dimensional structure of ScRD21A highlighting the catalytic triad (Cys-His-Asn) at the substrate-binding cleft.

Because RD21-like proteases are widely implicated in plant immunity and are frequent effector targets [17,21], we examined the sugarcane RD21 clade in greater detail. Multiple sequence alignment with *Arabidopsis* and rice orthologs showed strong conservation of the catalytic triad (Cys-His-Asn) and adjacent residues in the predicted substrate-binding region (Figure 3A). Among the sugarcane RD21-like members, SsRD21-4 and SsRD21-5 were the two closest homologs of Arabidopsis AtRD21A (Figure 1A). Based on BLASTP identity calculated as identical residues over the alignment length, SsRD21-4 showed higher identity to AtRD21A than SsRD21-5 (70.02% for SsRD21-4 versus 66.89% for SsRD21-5) (Figure S3). On this basis, SsRD21-4/Sspon.005D0003070 was selected as a representative sugarcane RD21-like candidate for downstream analysis and was designated ScRD21A.

AlphaFold3 modeling predicted that ScRD21A adopts the typical papain-like fold, with an L-domain enriched in α-helices and an R-domain dominated by antiparallel β-sheets (Figure 3B). The catalytic triad is positioned in the cleft between the two domains, with Cys156, His292, and Asn312 arranged in a geometry consistent with papain-family proteases (Figure 3B). This spatial arrangement is highly similar to that reported for biochemically characterized PLCPs, supporting the annotation of ScRD21A as a canonical papain-like cysteine protease [18].

### 2.4. SsPit2A/B Interact with ScRD21A in an LXRR-Dependent Manner

**Figure 4:**
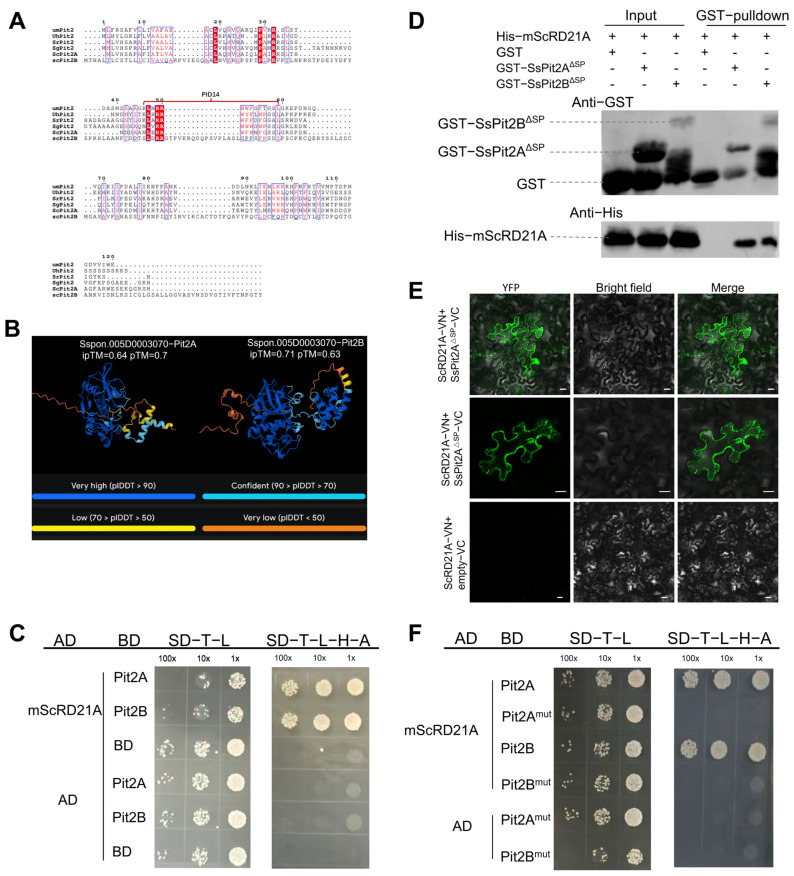
SsPit2A/B interact with ScRD21A in an LXRR-dependent manner. (**A**) Multiple sequence alignment of Pit2 homologs from *S. scitamineum* and other smut fungi (*Sporisorium reilianum*, *Sporisorium graminicola*, *Ustilago hordei*, *U. maydis*). Alignment was visualized with ESPript 3.0; red shading indicates strictly conserved residues. (**B**) AlphaFold3 predicted structure of the ScRD21A–SsPit2A/B interaction complex. pLDDT scores indicate per-residue local confidence; ipTM assesses interface reliability; pTM reflects overall complex folding accuracy. (**C**) Y2H assay showing interaction between the ScRD21A catalytic domain and SsPit2A/B. AD, GAL4 activation domain; BD, GAL4 DNA-binding domain; SD-T-L, synthetic dropout medium lacking Trp and Leu; SD-T-L-H-A, synthetic dropout medium lacking Trp, Leu, His, and Ade. (**D**) In vitro GST pull-down assay verifying direct binding of mature ScRD21A to SsPit2A/B. (**E**) BiFC assay showed ScRD21A interacts with SsPit2A/B in *N. benthamiana*. Scale bar, 20 μm. (**F**) Y2H analysis of interactions between ScRD21A and LXRR-mutated SsPit2A or SsPit2B proteins. Wild-type effectors supported growth on interaction-selective medium, whereas the corresponding LXRR-mutated variants did not. Combinations of Pit2A^mut^ or Pit2B^mut^ with the empty AD vector were included as negative controls.

PLCPs are key regulators of plant immunity and are recurrent targets of pathogen effectors that suppress host defense signaling [14,35]. We identified two Pit2-related sequences in *S. scitamineum*, designated SsPit2A and SsPit2B. SsPit2A represents the closest homolog to UmPit2, exhibiting the highest overall sequence similarity and a clearly conserved PID14 domain [36]. *SsPit2B* corresponds to the gene previously annotated as *SsPIT2* in a comparative expression study of high- and low-virulent *S. scitamineum* isolates [37]. Although SsPit2B shares moderate overall sequence identity with UmPit2, it possesses a PID14-like domain with notable conservation of key structural features, particularly the LXRR motif within this region (Figure 4A). We therefore selected SsPit2A and SsPit2B for further analysis. Sequence analysis revealed that SsPit2A harbors a canonical PID14 motif, whereas SsPit2B contains a structurally divergent PID14-like region with an insertion. Despite this variation, both proteins retain the conserved LXRR motif, which forms the functional core of the PID14 peptide and is essential for PLCP inhibition in other smut fungi [38].

We subsequently used AlphaFold3 to predict the complex structure between ScRD21A and the *S. scitamineum* effectors SsPit2A/B (Figure 4B). Model reliability was assessed by interface predicted template modeling score (ipTM) and predicted template modeling score (pTM). Both ScRD21A–SsPit2A and ScRD21A–SsPit2B pairs yielded ipTM values above 0.6, indicating a high probability of interaction, and pTM values above 0.5, supporting reliable overall complex folding. We further validated this interaction experimentally. In yeast two-hybrid (Y2H) assays, all co-transformants grew on SD/-Trp/-Leu medium (transformation control), whereas growth on SD/-Trp/-Leu/-His/-Ade was observed only for the ScRD21A–SsPit2A/B combinations, indicating interaction with the catalytic C1 domain of ScRD21A (Figure 4C). This interaction was further confirmed by GST pull-down assays, indicating direct physical association in vitro (Figure 4D). In planta, Bimolecular Fluorescence Complementation (BiFC) assays revealed robust complex formation between ScRD21A and SsPit2A/B, demonstrating that this interaction occurs in living plant cells (Figure 4E).

To identify the molecular determinant underlying this interaction, we mutated the basic amino acid residues within the conserved LXRR motif to alanine or glycine. Both wild-type SsPit2A and SsPit2B supported yeast growth on quadruple dropout medium, whereas the corresponding LXRR-mutated versions did not, indicating that the core LXRR motif is required for ScRD21A association (Figure 4F, Supplementary Table S4). Together, these data support an LXRR motif-dependent mode of ScRD21A targeting by SsPit2A and SsPit2B and are consistent with a substrate-mimicry model.

### 2.5. SsPit2A/B Co-Localize with ScRD21A and Are Associated with Its Subcellular Redistribution

**Figure 5:**
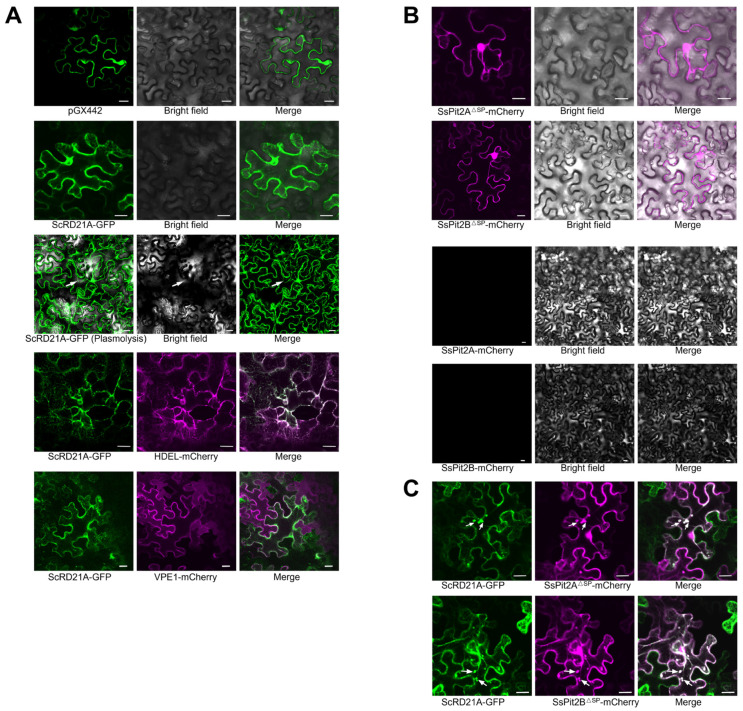
Co-localization analysis of SsPit2A/B and ScRD21A. (**A**) Subcellular localization of ScRD21A-GFP in *N. benthamiana* leaves. Plasmolysis was induced by treatment with 1 M mannitol for 10 min; white arrows indicate plasmolysis. Scale bar, 20 μm. (**B**) SsPit2A/B^ΔSP^-mCherry show nucleocytoplasmic distribution; full-length versions with native signal peptides are undetectable. Scale bar, 20 μm. (**C**) Co-expression of ScRD21A-GFP with SsPit2A/B^ΔSP^-mCherry produces cytoplasmic co-localization and punctate aggregates (white arrows). Scale bar, 20 μm. Green indicates GFP, magenta indicates mCherry, and their colocalization is shown in white.

The activity of RD21-like proteases is tightly linked to their subcellular localization and maturation state [18,39]. Previous studies place RD21-type PLCPs along the secretory–vacuolar route, including the endoplasmic reticulum (ER), vacuole, and apoplast [40]. Under non-stressed conditions, RD21 is largely sequestered in the vacuole and ER bodies, but vacuolar membrane disruption can release RD21 into the cytosol, where it promotes vacuole-driven cell death and defense-associated responses [41,42]. To determine the localization of ScRD21A, we transiently expressed ScRD21A-GFP in *N. benthamiana*. ScRD21A localized predominantly to intracellular compartments and showed no detectable apoplastic signal following plasmolysis, indicating retention within the endomembrane system (Figure 5A). Co-localization analysis revealed partial overlap with both ER and vacuolar markers, consistent with the canonical secretory–vacuolar trafficking route of RD21 proteases (Figure 5A). This localization pattern is compatible with a model in which ScRD21A undergoes maturation within vacuolar compartments before activation.

Most predicted *S. scitamineum* secreted effectors encode an N-terminal signal peptide [28]. For transient expression assays, signal peptide-deleted constructs are commonly used to retain proteins intracellularly and improve detection [43]. When signal peptide-deleted SsPit2A/B-mCherry fusions were transiently expressed in *N. benthamiana*, fluorescence was detected in both the nucleus and cytoplasm, whereas the full-length constructs yielded no detectable intracellular signal (Figure 5B). Because the predicted signal peptides are expected to route the full-length fusions into the secretory pathway, the lack of detectable intracellular fluorescence may reflect secretion-associated low accumulation, extracellular instability, proteolytic processing, or reduced mCherry stability after secretion. Thus, the SsPit2A/B^ΔSP^-mCherry fusions were used in subsequent localization and co-expression assays as predicted mature effector forms. Strikingly, co-expression of SsPit2A/B^ΔSP^ induced a pronounced relocalization of ScRD21A from a diffuse intracellular distribution to discrete cytoplasmic puncta, suggesting altered trafficking or compartmentalization (Figure 5C). Effector-mediated relocalization of host proteins is an emerging mechanism for suppressing plant immunity [44]. Given that RD21 activation depends on proper trafficking and proteolytic processing, the redistribution of ScRD21A observed here likely impacts its maturation state or substrate accessibility. These findings indicate that SsPit2A and SsPit2B not only interact with ScRD21A but may also be associated with changes in its spatial distribution within plant cells.

### 2.6. SsPit2A/B Reduce ScRD21A-Associated Cysteine Protease Activity, Whereas ScRD21A Supports Defense-Related Responses

**Figure 6:**
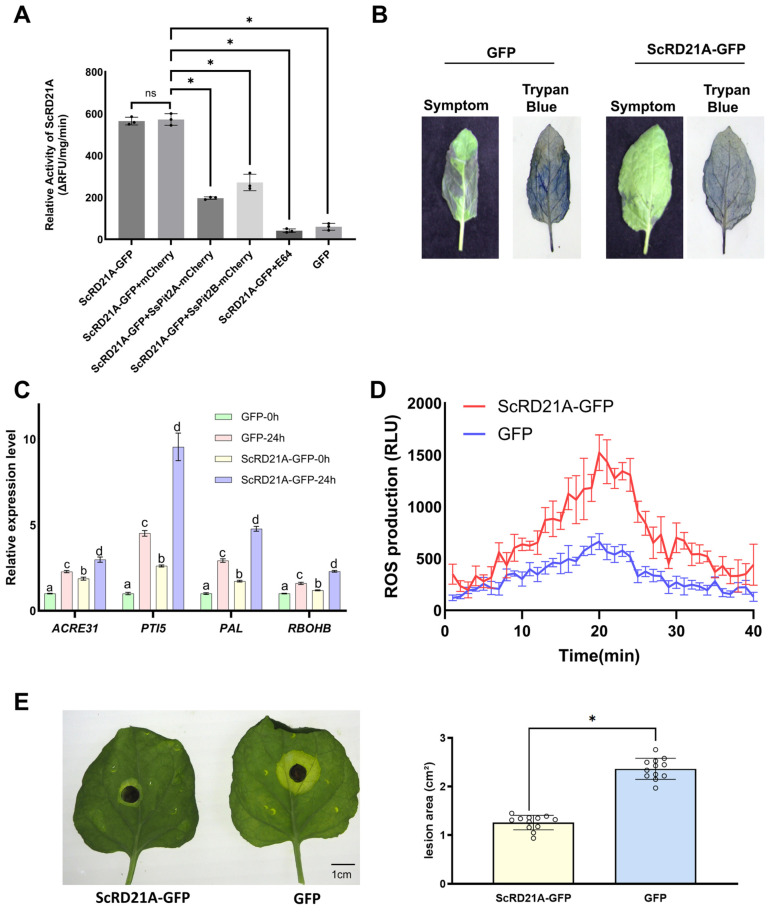
Analysis of ScRD21A cysteine protease activity in the presence of SsPit2A/B and evaluation of ScRD21A-associated defense phenotypes. (**A**) Assessment of ScRD21A-associated enzymatic activity in the presence of SsPit2A/B. Total protein was extracted from *N. benthamiana* leaves 48 h after transient expression, activity was measured in vitro using the fluorogenic substrate N-CBZ-Phe-Arg-AMC and expressed as ΔRFU mg^−1^ min^−1^. Data are shown as means ± SD, n = 3. One-way ANOVA with Dunnett’s post hoc test (* *p* < 0.05). (**B**) Trypan blue staining of leaves infiltrated with *Pst* DC3000 at 48 hpi, revealing reduced hypersensitive cell death in ScRD21A-expressing leaves compared to controls. (**C**) Expression of defense-related genes before (0 h) and 24 h after *Pst* DC3000 inoculation. Transcript levels of representative immunity genes were quantified by qRT-PCR using NbEF1α as internal reference. Data are normalized to the 0 h control (set to 1). Data are shown as means ± SD, n = 3; Different letters denote significant differences (*p* < 0.05) using Tukey’s multiple comparisons test after two-way ANOVA. (**D**) Dynamic ROS production in leaf disks after flg22 elicitation. Leaf disks from *N. benthamiana* transiently expressing ScRD21A-GFP or GFP control were incubated in luminol assay solution; relative light units (RLU) were recorded continuously for 40 min. Data are shown as mean RLU ± SD (n = 8–12 disks per group). (**E**) Detached-leaf assay with *B. cinerea*. Leaves expressing ScRD21A-GFP or GFP control (48 h post-infiltration) were inoculated with 6 mm mycelial plugs. Lesion diameters were measured after 72 h at 22 °C under high humidity. Bars = mean ± SD (n = 10–15). * *p* < 0.05 vs. GFP control (Student’s *t*-test).

To determine the functional consequence of SsPit2A/B–ScRD21A interaction, we measured protease activity using a fluorogenic substrate. Co-expression with SsPit2A or SsPit2B significantly reduced the measured cysteine protease activity in total protein extracts to 49.2% and 35.3% of control levels, respectively (Figure 6A). The sensitivity of the assay to E-64 confirmed that the measured activity corresponds to cysteine proteases. Protease inhibition is a hallmark virulence strategy, particularly for PLCPs that function as immune regulators [14,45]. Although the use of crude extracts from transiently expressing *N. benthamiana* tissue and a generic fluorogenic substrate does not by itself establish direct biochemical inhibition of purified ScRD21A, the significant reduction in E-64-sensitive activity upon SsPit2A/B co-expression supports the conclusion that SsPit2A/B attenuate ScRD21A-associated cysteine protease activity in this system.

To evaluate the role of ScRD21A in plant immunity, we employed the well-established *Pseudomonas syringae pv. tomato* (*Pst*) DC3000–*N. benthamiana* pathosystem, which triggers a robust hypersensitive response (HR). At 48 h post-inoculation with *Pst* DC3000, trypan blue staining revealed extensive deep-blue necrotic lesions in GFP control leaves, indicative of severe cell death. In contrast, leaves transiently overexpressing ScRD21A-GFP exhibited markedly reduced necrotic areas, suggesting that ScRD21A attenuates pathogen-induced hypersensitive cell death (Figure 6B).

To further assess downstream transcriptional responses, we quantified the expression of defense-associated marker genes. qRT-PCR analysis revealed significantly higher transcript levels of the PTI marker genes *ACRE31* and *PTI5*, the ROS-producing gene *RBOHB*, and the salicylic acid (SA) pathway gene *PAL* in ScRD21A-GFP-expressing leaves than in GFP controls (Figure 6C). Notably, all four genes exhibited elevated basal expression in ScRD21A-GFP leaves even before inoculation, with their induction remaining substantially stronger following pathogen challenge. Upon flg22 treatment, leaves expressing ScRD21A displayed a stronger and more rapid ROS burst compared with control tissues (Figure 6D). This enhanced ROS production was characterized by both a higher peak amplitude and accelerated induction kinetics, indicating that ScRD21A contributes to the amplification of early immune signaling.

In detached-leaf assays with *B. cinerea*, lesion areas in ScRD21A-GFP-expressing leaves were markedly smaller than in controls at 72 h post-inoculation (Figure 6E), suggesting that ScRD21A also enhances resistance to this necrotrophic fungal pathogen. Together, these results establish ScRD21A as a positive regulator of plant immunity.

## 3. Discussion

PLCPs are important components of plant immune regulation and are frequent targets of pathogen effectors [14,15]. In this study, we combined genome-wide characterization of the sugarcane PLCP family with functional analysis of an effector–protease module relevant to sugarcane smut. We identified 61 ScPLCP genes in the *S. spontaneum* AP85-441 genome, defined their major structural and evolutionary features, and selected the RD21-like protease ScRD21A for downstream analysis. We further identified two effectors from sugarcane smut, SsPit2A and SsPit2B, and showed that they associate with ScRD21A in an LXRR-dependent manner and reduce ScRD21A-associated cysteine protease activity. We also analyzed the co-localization of ScRD21A with SsPit2A/B, while functional assays supported a positive role for ScRD21A in defense-related responses. Together, these findings provide a framework for understanding effector–PLCP interactions in the sugarcane–*S. scitamineum* pathosystem.

ScRD21A was prioritized because RD21-like proteases occupy a particularly relevant position at the interface between host defense and pathogen virulence, and because ScRD21A/SsRD21-4 showed the highest sequence identity to AtRD21A among the sugarcane RD21-like candidates examined. In Arabidopsis and other systems, RD21-family members contribute to resistance-associated proteolysis and are recurrent targets of pathogen interference [15,19,20,21,22,23,39]. The conservation of the catalytic triad and the overall papain-like fold in ScRD21A therefore supports its annotation as a plausible immune-relevant protease in sugarcane. Given the size of the sugarcane PLCP family and the complexity of the sugarcane genome, ScRD21A is best viewed as a representative entry point for investigating defense-relevant PLCPs in this pathosystem, rather than as the only PLCP likely to contribute to host defense.

We identified two Pit2 homologs in *S. scitamineum*, and both SsPit2A and SsPit2B interact with ScRD21A. This observation is important because Pit2 represents one of the best characterized PLCP targeting effectors in smut fungi. In the *U. maydis*–maize pathosystem, Pit2 is processed by host proteases to release a short inhibitory region, and a conserved motif within this region is essential for suppression of host PLCP activity [38,45]. Our data suggest that a related mode of PLCP targeting may also operate in the sugarcane smut fungus. In particular, the mutational results indicate that the PID14-like region, especially the embedded LXRR motif, is important for ScRD21A targeting by SsPit2A/B. Although SsPit2B contains an insertion relative to the canonical PID14 motif and the two proteins differ substantially outside this region, the loss of interaction caused by LXRR mutation suggests that this motif may be more critical than the surrounding sequence context for maintaining PLCP-directed activity. This interpretation is consistent with the idea that smut effectors can diversify extensively at the sequence level while retaining short functional elements required for host target engagement [15]. Whether the two *S. scitamineum* Pit2 paralogs differ in inhibitory capacity, or infection-stage regulation remains unresolved and will require direct testing in the native pathosystem.

Functional assays further showed that SsPit2A and SsPit2B reduce ScRD21A-associated protease activity. Because the fluorogenic assay was performed using total protein extracts from transiently expressing *N. benthamiana* tissue, the reduced substrate turnover should be interpreted with appropriate caution and may reflect the integrated consequences of effector association with ScRD21A in planta rather than a fully resolved biochemical mechanism. Nevertheless, the combination of pull-down evidence, interaction in planta, and the dependence on the conserved LXRR motif strongly supports effector-associated attenuation of ScRD21A-related protease output. These findings place SsPit2A/B within an expanding set of RD21-targeting effectors and support an RD21-directed virulence strategy in the sugarcane smut fungus, although the precise biochemical mechanism will require further resolution [14,15].

In addition, we observed that co-expression with SsPit2A/B was associated with changes in the subcellular distribution pattern of ScRD21A, suggesting that these effectors may also influence the localization of the host protease. This aspect is mechanistically intriguing because RD21-like proteases are tightly linked to endomembrane trafficking, maturation, and compartment-specific activation. Endogenous regulators such as Serpin1 and the deubiquitinase OTU2 illustrate that RD21 activity and abundance are under multilayered control even in the absence of pathogens [22,40]. The punctate co-localization patterns observed here are consistent with altered trafficking or maturation of ScRD21A, but the available data do not yet support a definitive conclusion. Because the localization experiments used signal-peptide-deleted effectors in a heterologous system, the puncta could represent trafficking intermediates, aberrant aggregates, or host compartments not resolved by the current markers. In the classical secretory pathway, N-terminal signal peptides are generally cleaved during ER translocation, and the *U. maydis* Pit2 effector has been discussed as a mature protein after signal peptide cleavage [38]. Thus, the ΔSP constructs used here likely represent the predicted mature SsPit2A/B forms. The absence of detectable fluorescence from full-length SsPit2A/B-mCherry may reflect weak apoplastic accumulation, extracellular instability, proteolytic processing, or reduced mCherry maturation/stability after secretion. Therefore, these assays support the behavior of the predicted mature effector forms but do not directly demonstrate secretion of full-length SsPit2A/B during infection. Defining the identity of these structures, and testing whether protease activity or the LXRR motif is required for their formation, will be important next steps.

In the *N. benthamiana* transient expression system, ScRD21A enhanced flg22-triggered ROS burst, attenuated *Pst DC3000*-induced cell death, and increased resistance to *B. cinerea*. Although these assays do not reproduce the native sugarcane-smut interaction, they consistently support a positive contribution of ScRD21A to plant immunity. This pattern is consistent with evidence that PLCPs do not function solely as terminal degradative enzymes, but can also shape immune signaling outputs more broadly. For example, the XCP1-CYSTATIN6 module affects pattern-triggered immunity by influencing RBOHD stability, and RD21-related proteolysis can release bioactive peptides that reinforce host defense [19,46]. Our data do not yet reveal whether ScRD21A acts through direct antimicrobial proteolysis, processing of endogenous immune signals, regulation of protein turnover along the secretory-vacuolar route, or a combination of these mechanisms. Nevertheless, the elevated basal and inducible expression of defense-associated marker genes together with the stronger flg22-triggered ROS burst are consistent with the interpretation that ScRD21A contributes positively to immune responses rather than merely altering tissue damage.

However, because the functional analyses in this study were performed in a heterologous system rather than in sugarcane, the precise contribution of ScRD21A to smut resistance still warrants validation in the native host. This point should be considered in the context of the highly polyploid and genetically complex sugarcane genome, in which functional overlap among PLCP family members is likely [1]. In parallel, although only a limited number of *S. scitamineum* effectors have been experimentally characterized to date, the available studies already indicate that this pathogen deploys multiple virulence strategies, including peptide mimicry and manipulation of host trafficking [28,32,47]. Within this context, the ScRD21A–SsPit2A/B module offers a practical entry point for dissecting effector–protease interactions during sugarcane smut infection. From an applied perspective, SsPit2A/B could be used as molecular probes to screen sugarcane germplasm for RD21-like PLCP alleles that show reduced effector association while retaining protease function. ScRD21A may also serve as a candidate host factor for testing whether RD21-like protein abundance, processing, trafficking, effector sensitivity, or protease output correlates with smut tolerance in resistant and susceptible sugarcane materials. These possibilities remain provisional and will require validation in sugarcane germplasm and infection assays. Future work should prioritize direct validation of ScRD21A in sugarcane and determine whether SsPit2A/B affect ScRD21A accumulation or maturation and whether LXRR mutation compromises their inhibitory activity in the native host. Clarifying whether ScRD21A functions through immune-relevant substrates or defense-active peptides will further refine its role in this interaction [19]. These analyses will also help assess whether RD21-related pathways could support resistance improvement [22,48]. Overall, the ScRD21A–SsPit2A/B module provides a testable model for understanding sugarcane smut pathogenesis and for future host-directed validation.

## 4. Materials and Methods

### 4.1. Genome-Wide Analysis of the ScPLCP Family

Genome-wide identification of the PLCP family in sugarcane was performed using the monoploid genome of *S. spontaneum* AP85-441 available in the Sugarcane Genome Database (ScDB; http://sugarcane.gxu.edu.cn/scdb/ (accessed on 13 May 2025)) [1]. The Hidden Markov Model (HMM) profile of the papain family cysteine protease domain (PF00112) was employed to search the sugarcane protein database with HMMER 3.0 (E-value cutoff < 1 × 10^−5^) [49]. The representative HMMER command used to retrieve candidate PLCPs as Supplementary Information (Supplementary Table S2). Candidate sequences were further validated by BLASTP searches (E-value < 1 × 10^−5^) against well-characterized PLCP sequences from *Arabidopsis* Col-0 and rice Nipponbare, retrieved from TAIR (https://www.arabidopsis.org (accessed on 13 May 2025)) and the Rice Genome Annotation Project (http://rice.uga.edu (accessed on 13 May 2025)), respectively [18,34,50]. Redundant sequences were removed, resulting in the identification of 61 non-redundant ScPLCP genes.

Physicochemical properties of the deduced ScPLCP proteins, including molecular weight (MW), isoelectric point (pI), amino acid length, grand average of hydropathicity (GRAVY), and instability index, were calculated using the ExPASy ProtParam tool (https://web.expasy.org/protparam/ (accessed on 13 May 2025)) [51]. Multiple sequence alignment was conducted with Clustal Omega. A phylogenetic tree was constructed using MEGA X with the Neighbor-Joining method based on the Jones-Taylor-Thornton (JTT) model and 1000 bootstrap replicates; branches with bootstrap support > 50 were considered reliable [52]. Maximum likelihood (ML) phylogenetic analysis was performed using IQ-TREE via the TBtools Phylogenetics module (IQ-tree Wrapper plugin). The tree was visualized and annotated using iTOL (https://itol.embl.de/ (accessed on 13 May 2025)) [53], allowing classification of ScPLCPs into nine subfamilies.

Pairwise protein similarity matrices were generated using TBtools v2.0 (Protein Pairwise Similarity Matrix module, default gap penalties) [54]. Conserved domains were identified using the NCBI Conserved Domain Search (CD-Search) tool. Conserved motifs were analyzed with the MEME Suite [55]. Chromosomal locations of ScPLCP genes were mapped using the Circos module in TBtools based on the corresponding GFF3 annotation file. Synteny analysis between *S. spontaneum* AP85-441 and *O. sativa* (Nipponbare) was performed with MCScanX [56].

To investigate potential regulatory elements, 2000 bp upstream promoter sequences of *ScPLCP* genes were extracted and analyzed for cis-acting regulatory elements using PlantCARE (https://bioinformatics.psb.ugent.be/webtools/plantcare/html/ (accessed on 13 May 2025)) [57]. Elements were classified according to their predicted functions (e.g., hormone-responsive: ABA, SA; stress-responsive: pathogen, drought; developmental: meristem-specific) and visualized with TBtools.

The ScPLCP member showing the highest sequence similarity to AtRD21A (Sspon.005D0003070, hereafter designated ScRD21A) was selected for structural modeling. Its three-dimensional structure was predicted using the AlphaFold3 online server (https://alphafoldserver.com/ (accessed on 13 May 2025)) [58]. Model confidence was evaluated using the confidence metrics reported by AlphaFold3, including per-residue pLDDT values and overall pTM/ipTM scores where applicable. Structural visualization and comparison of the conserved catalytic triad (Cys-His-Asn) were performed using UCSF ChimeraX 1.8 [59]. AlphaFold3-multimer modeling was performed to predict effector–protease interaction interfaces [58].

### 4.2. Subcellular Localization

Constructs for transient expression (35S::ScRD21A-GFP, 35S::mCherry, and 35S::SsPit2A/B-mCherry) were introduced into *Agrobacterium tumefaciens* strain GV3101. Overnight cultures were resuspended in infiltration buffer (10 mM MES, 10 mM MgCl_2_, 150 μM acetosyringone, pH 5.6) and adjusted to an OD_600_ of approximately 0.6. The suspensions were infiltrated into the abaxial surface of fully expanded leaves of 4-week-old *N. benthamiana* plants. Fluorescence signals were observed 48 h post-infiltration using an Olympus FV3000 confocal laser scanning microscope (Olympus Corporation, Tokyo, Japan) with appropriate excitation/emission settings for GFP and mCherry.

### 4.3. Y2H

Y2H assays were conducted essentially as described previously [60]. The C1 protease domain of ScRD21A and full-length fragments of SsPit2A/B were cloned into the pGADT7 (prey) and pGBKT7 (bait) vectors, respectively. Constructs were co-transformed into yeast strain Y2HGold. Transformants were first selected on SD/-Trp/-Leu medium (SD-T-L) and then assayed on SD/-Trp/-Leu/-His/-Ade medium (SD-T-L-H-A) (Takara Bio USA, Inc., San Jose, CA, USA). Empty vectors served as negative controls. Site-directed mutagenesis was used to generate LXRR motif mutants of SsPit2A/B to evaluate their contribution to the interaction.

### 4.4. BiFC

BiFC assays were performed following established protocols [60] with minor modifications. For ScRD21A, the coding sequence of the mature protein was amplified; for SsPit2A and SsPit2B, the coding sequences devoid of the signal peptide (ΔSP) were amplified. The resulting fragments were cloned into the pDOE-03 vector, a single-vector dual-ORF expression system designed for BiFC analysis, via the In-Fusion cloning strategy. Recombinant constructs were transformed into *A. tumefaciens* strain GV3101 and co-infiltrated into *N. benthamiana* leaves as described in Section 4.2. At 48 h post-infiltration, YFP fluorescence was visualized using an Olympus FV3000 confocal laser scanning microscope under the following acquisition parameters: excitation at 488 nm and emission collection between 520 and 560 nm. As a negative control, an empty VC cassette was incorporated into the same pDOE-03 backbone alongside the ScRD21A-VN fusion cassette. For each experimental combination, a minimum of three independently infiltrated leaves were examined, with three to five fields of view captured per leaf.

### 4.5. Protease Activity Assays

Total proteins were extracted from *N. benthamiana* leaves 48 h post-infiltration and quantified by BCA assay. Cysteine protease activity was measured fluorometrically using the substrate N-CBZ-Phe-Arg-AMC (Aladdin, Shanghai, China). The 50 μL reaction mixture contained 40 μL crude protein extract and 10 μL of 1 mM N-CBZ-Phe-Arg-AMC. Reactions were incubated in the dark for 10 min at 25 °C, after which fluorescence intensity was monitored (excitation 380 nm, emission 460 nm) using a BioTek Synergy H1 multimode reader (BioTek Instruments, Inc., Winooski, VT, USA) [21]. E-64 was included as an inhibitor control to verify cysteine-protease-dependent activity. Enzymatic activity was normalized to total protein abundance and expressed as ΔRFU/mg/min. Because total extracts and a generic substrate were used, the measured values are interpreted as ScRD21A-associated cysteine protease output rather than direct activity of purified ScRD21A. All assays were performed in three independent biological replicates, and results are presented as means ± standard deviation.

### 4.6. Disease-Related Assays

Transient overexpression in *N. benthamiana* was achieved via *A. tumefaciens* GV3101 infiltration as detailed in Section 4.2. For ROS burst, *Pst* DC3000 challenge, defense-gene expression, and *B. cinerea* assays, leaves were infiltrated with 35S::ScRD21A-GFP or GFP control only; SsPit2A/B constructs were not used in these assays. Assays were conducted at the indicated time points post-infiltration.

For ROS burst assays, *N. benthamiana* leaves were agroinfiltrated and incubated for 48 h. Leaf disks were floated in water overnight and then treated with 100 nM flg22, 100 μM luminol, and 20 μg mL^−1^ horseradish peroxidase. Luminescence was measured every 1 min for 40 min using a BioTek Synergy H1 multimode reader [61].

At 24 h post-agroinfiltration, *N. benthamiana* leaves were inoculated with *Pst* DC3000 at OD_600_ = 0.3. Defense-related gene expression was analyzed by qRT-PCR at 24 hpi with *Pst* DC3000. Cell death was visualized by trypan blue staining at 48 hpi with *Pst* DC3000. Cell death was visualized by trypan blue staining at 48 h post-inoculation with *Pst* DC3000 [62]. For defense-gene expression analysis, total RNA was isolated from the indicated leaf tissues and reverse transcribed for qRT-PCR. Relative transcript levels were normalized to *NbEF1α* and calculated against the 0 h control. Unless otherwise indicated, primer pairs used for transcript analysis are listed in the Supplementary Materials.

For *B. cinerea* infection assays, 6 mm mycelial plugs were placed on wounded leaves at 48 h after agro-infiltration, and lesion areas were measured 72 h later under high humidity at 22 °C [63].

Statistical analyses were performed using the tests specified for each experiment, including one-way ANOVA with Dunnett’s post hoc test, two-way ANOVA for multi-factor comparisons, and Student’s *t*-test for pairwise comparisons. Differences were considered significant at *p* < 0.05.

### 4.7. Pull-Down Assays

His-mScRD21A and GST-SsPit2A/B^ΔSP^ fusion proteins were expressed in *Escherichia coli* BL21 following induction with 1 mM IPTG at 16 °C for 16 h. GST-tagged bait proteins were immobilized onto glutathione-agarose beads (Thermo Fisher Scientific, Waltham, MA, USA) by incubation at 4 °C for 1–2 h with gentle rotation. The beads were subsequently washed three times with ice-cold binding buffer (20 mM Tris-HCl, pH 7.5; 150 mM NaCl; 0.5–1% Triton X-100; 1 mM EDTA; 1 mM DTT; 1 mM MgCl_2_) to remove unbound proteins, then blocked with 2–3 mg/mL BSA in wash buffer at 4 °C for 30–60 min. Following a brief wash, the bead-bound GST fusions were incubated with purified His-mScRD21A prey protein at 4 °C for 1–2 h with gentle rotation. The beads were extensively washed with ice-cold wash buffer (20 mM Tris-HCl, pH 7.5; 250–300 mM NaCl; 0.5–1% Triton X-100; 1 mM EDTA; 1 mM DTT; 1 mM MgCl_2_) to eliminate non-specifically bound proteins. Bound proteins were eluted by boiling in 1× SDS loading buffer at 95–100 °C for 5–10 min. The eluates were subjected to SDS-PAGE and analyzed by Western blot using anti-His (EasyBio, Hangzhou, China) and anti-GST (TransGen Biotech, Beijing, China) antibodies. GST alone served as a negative control throughout the assay.

## Figures and Tables

**Table 1:** Summary of sugarcane ScPLCP family members including gene IDs, sequence length, isoelectric point, molecular weight, and hydrophobicity index.

Subfamily	Gene ID	Sequence ID	Number of Amino Acid	Molecular Weight (Da)	Theoretical pI	Instability Index	Aliphatic Index	Grand Average of Hydropathicity
AALP	*AALP-1*	Sspon.002D0014060	377	41,001.36	6.29	31.3	72.23	−0.274
	*AALP-2*	Sspon.002C0018051	354	38,364.43	8.3	21.53	76.98	−0.127
	*AALP-3*	Sspon.002B0014540	387	42,083.68	8.15	29.16	75.19	−0.145
	*AALP-4*	Sspon.002A0017790	352	37,873.63	5.59	28.22	78.49	−0.076
CEP	*CEP-1*	Sspon.006A0020700	358	39,878.81	6.17	45.33	71.17	−0.463
	*CEP-10*	Sspon.003B0004920	369	40,474.19	6.14	39.52	70.08	−0.413
	*CEP-11*	Sspon.006D0024940	364	40,728.87	5.11	29.83	71.48	−0.448
	*CEP-2*	Sspon.006D0019340	367	40,754.66	6.09	44.29	71.61	−0.44
	*CEP-3*	Sspon.006C0023300	340	37,833.34	7.6	42.81	64.32	−0.627
	*CEP-4*	Sspon.006C0023340	723	79,608.89	7.32	49.83	87.58	−0.161
	*CEP-5*	Sspon.006C0005051	372	40,301.23	6.28	34	70.16	−0.348
	*CEP-6*	Sspon.006A0005571	370	40,116.99	6.14	33.5	70.03	−0.35
	*CEP-7*	Sspon.001B0042910	375	40,880.64	6.36	37.1	69.73	−0.392
	*CEP-8*	Sspon.001C0011080	375	40,994.74	6.5	39.53	68.93	−0.422
	*CEP-9*	Sspon.001D0039510	375	40,880.64	6.36	37.1	69.73	−0.392
CTB	*CTB-1*	Sspon.002C0010060	336	36,571.1	6.39	34.02	88.45	0.019
RD19	*RD19-1*	Sspon.005D0018591	375	41,229.39	6.16	32.3	74.13	−0.355
	*RD19-2*	Sspon.005C0013841	376	41,282.45	6.16	32.58	74.97	−0.354
	*RD19-3*	Sspon.005A0016621	377	41,499.76	6.16	30.92	75.81	−0.334
	*RD19-4*	Sspon.005B0014541	177	19,410.99	7.72	22.58	70.96	−0.321
	*RD19-5*	Sspon.004D0016796	299	32,207.26	5.43	24.12	75.69	−0.209
	*RD19-6*	Sspon.002A0010010	398	42,444.38	9.74	45.54	77.64	−0.167
RD21	*RD21-1*	Sspon.005A0002090	465	50,182.63	5.67	34.09	70.75	−0.269
	*RD21-2*	Sspon.005D0001320	465	50,194.62	5.67	34.33	71.38	−0.27
	*RD21-3*	Sspon.005A0002670	463	50,078.46	6.6	36.66	69.59	−0.355
	*RD21-4*	Sspon.005D0003070	464	50,311.75	4.95	36.08	62.95	−0.465
	*RD21-5*	Sspon.005A0003210	480	52,240.9	4.69	34.25	67.75	−0.393
	*RD21-6*	Sspon.008D0003331	1533	165,423.4	5.08	34.05	78.49	−0.22
	*RD21-7*	Sspon.008A0004100	343	37,453.75	4.82	32.95	79.13	−0.31
	*RD21-8*	Sspon.008A0004081	375	40,869.83	5.66	31.56	76.03	−0.396
	*RD21-9*	Sspon.008B0003190	399	43,447.75	5.23	33.65	76.32	−0.307
SAG12	*SAG12-1*	Sspon.008B0013101	354	38,429.41	5.44	23.43	68.42	−0.247
	*SAG12-2*	Sspon.008D0013320	301	32,470.49	5.52	17.7	68.14	−0.292
	*SAG12-3*	Sspon.008D0012401	339	36,747.47	5.39	19.42	65.96	−0.259
	*SAG12-4*	Sspon.008B0013130	359	39,029.97	5.4	23.19	68.27	−0.303
	*SAG12-5*	Sspon.008A0012920	320	34,685.09	5.28	17.96	68.66	−0.265
	*SAG12-6*	Sspon.008B0013141	339	36,732.39	5.26	16.34	67.14	−0.292
	*SAG12-7*	Sspon.008A0012960	319	34,358.78	5.36	20.22	65.55	−0.244
	*SAG12-8*	Sspon.008B0013100	338	36,563.24	6.03	19.97	66.15	−0.293
	*SAG12-9*	Sspon.008C0011742	304	32,785.91	5.26	19.2	61.71	−0.325
	*SAG12-10*	Sspon.005A0018151	339	36,641.74	5.74	24.88	70.27	−0.167
	*SAG12-11*	Sspon.005C0015020	324	35,219	5.22	21.68	75.31	−0.13
	*SAG12-12*	Sspon.005D0019660	298	32,560.8	5.57	22.25	64.83	−0.389
	*SAG12-13*	Sspon.005C0015000	343	36,822.72	4.69	29.19	63.82	−0.328
	*SAG12-14*	Sspon.001A0035091	353	37,484.68	5.54	35.71	69.86	−0.248
	*SAG12-15*	Sspon.006B0018500	347	37,043.16	4.82	23.5	69.83	−0.199
THI	*THI-1*	Sspon.002D0011310	351	37,619.52	6.15	30.51	73.68	−0.198
	*THI-2*	Sspon.002C0015740	369	39,876.19	5.41	28.35	79.02	−0.131
	*THI-3*	Sspon.002A0014560	351	37,838.85	5.65	31.81	75.36	−0.202
	*THI-4*	Sspon.002C0024610	346	37,097.28	4.91	37.73	69.74	−0.35
	*THI-5*	Sspon.002A0024470	381	40,719.49	5.4	40.43	71.29	−0.292
	*THI-6*	Sspon.002B0004550	360	39,585.63	7.69	39.25	71.56	−0.452
XBCP	*XBCP-1*	Sspon.007A0001083	453	47,750.32	6.75	40.62	77.7	−0.042
	*XBCP-2*	Sspon.007B0005760	448	47,737.64	8.55	37.97	88.33	−0.009
	*XBCP-3*	Sspon.007C0003450	458	48,455.86	6.16	43.03	69.15	−0.189
XCP	*XCP-1*	Sspon.007B0020900	374	40,745.38	5.19	33.47	70.72	−0.389
	*XCP-2*	Sspon.007D0018810	374	40,757.43	5.19	32.95	71.5	−0.374
	*XCP-3*	Sspon.003C0002300	376	40,886.5	5.22	39.09	73.43	−0.405
	*XCP-4*	Sspon.003C0002430	369	40,133.68	5.27	39.42	74.04	−0.396
	*XCP-5*	Sspon.003B0000291	377	40,783.37	5.56	40.27	69.89	−0.418
	*XCP-6*	Sspon.004C0006231	356	39,289.5	6.51	32.28	72.89	−0.264

## Data Availability

The original contributions presented in this study are included in the article. Further inquiries can be directed to the corresponding authors.

## Supplementary Materials

The following supporting information can be downloaded at: https://www.mdpi.com/article/10.3390/plants15091408/s1, Table S1: Primer sequences used in this study; Table S2: Representative HMMER command and filtering criteria used for PLCP retrieval; Table S3: MEME motif information associated with the eight conserved ScPLCP motifs. Table S4: Mutation sites and amino acid changes introduced in SsPit2A and SsPit2B. Figure S1: Neighbor-joining phylogenetic tree of PLCP proteins from sugarcane (*S. spontaneum* AP85-441), *Arabidopsis thaliana* (ecotype Columbia-0) and *Oryza sativa* ssp. japonica cv. *Nipponbare.* Subfamily-specific clades are color-coded. Figure S2: Pairwise protein sequence similarity matrix of ScPLCPs visualized using TBtools; Figure S3: Multiple sequence alignment of SsRD21-4 (Sspon.005D0003070), SsRD21-5 (Sspon.005A0003210) and AtRD21A (AT1G47128) from sugarcane and Arabidopsis, generated with MEGA and visualized using ESPript 3.0.

## Abbreviations

The following abbreviations are used in this manuscript:

ABA	Abscisic acid
BiFC	Bimolecular fluorescence complementation
ER	Endoplasmic reticulum
ETI	Effector-triggered immunity
flg22	22-amino-acid epitope of bacterial flagellin
HR	Hypersensitive response
ipTM	Interface predicted Template Modeling score
MeJA	Methyl jasmonate
PAMP	Pathogen-associated molecular pattern
PCD	Programmed cell death
PID14	Pit2 inhibitory domain 14
pTM	Predicted Template Modeling score
PLCP	Papain-like cysteine protease
PRR	Pattern recognition receptor
PTI	Pattern-triggered immunity
RBOHD	NADPH oxidase RESPIRATORY BURST OXIDASE HOMOLOG D
ROS	Reactive oxygen species
SA	Salicylic acid
SAR	Systemic acquired resistance
Y2H	Yeast two-hybrid
