# Supplementary material for: SsPit2A/B Effectors from Sporisorium scitamineum Interact with the Sugarcane PLCP ScRD21A and Reduce ScRD21A-Associated Cysteine Protease Activity via a Conserved LXRR Motif
Source: Plants (Basel). 2026 May 5;15(9):1408. doi: 10.3390/plants15091408 (PMC13165106; doi:10.3390/plants15091408)
Supplement: Supplementary file 1 [file plants-15-01408-s001.zip › Supplementary Materials/Supplementary Figure.pdf]

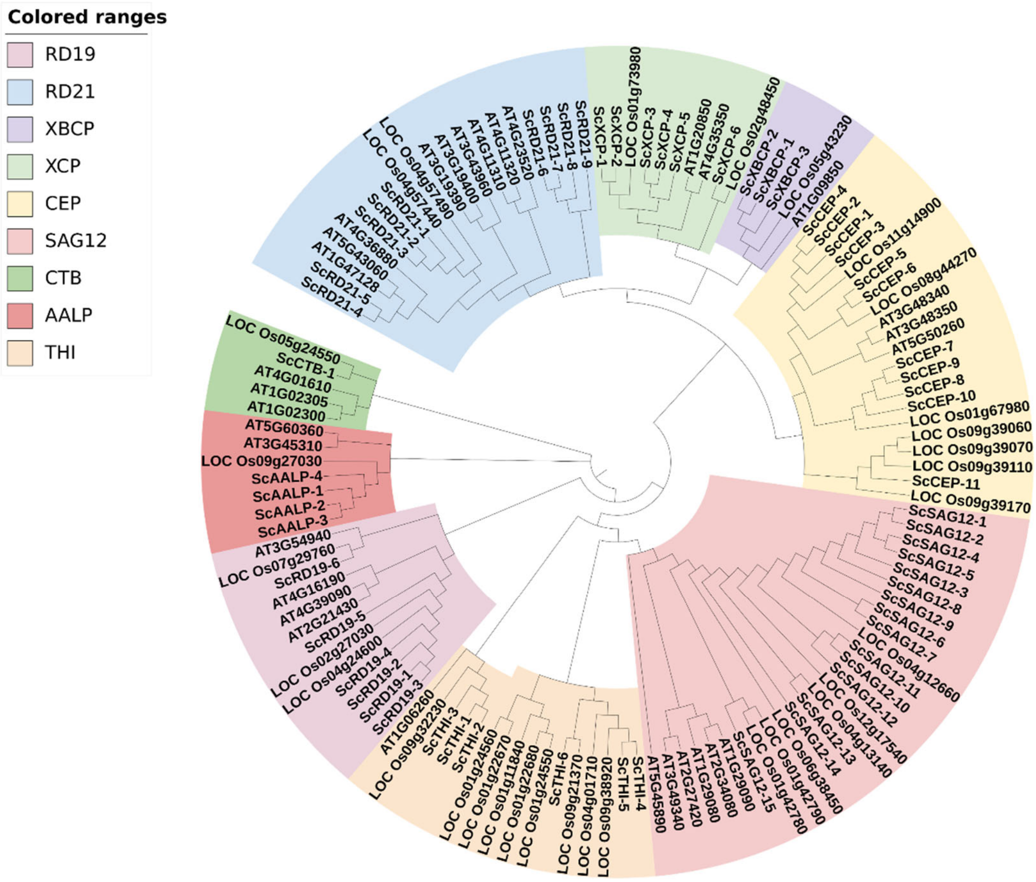

Figure S1. Neighbor-joining (NJ) phylogenetic tree of PLCP proteins from sugarcane (*S. spontaneum* AP85-441), *Arabidopsis thaliana* (ecotype Columbia-0) and *Oryza sativa* ssp. *japonica* cv. Nipponbare. Subfamily-specific clades are color-coded.

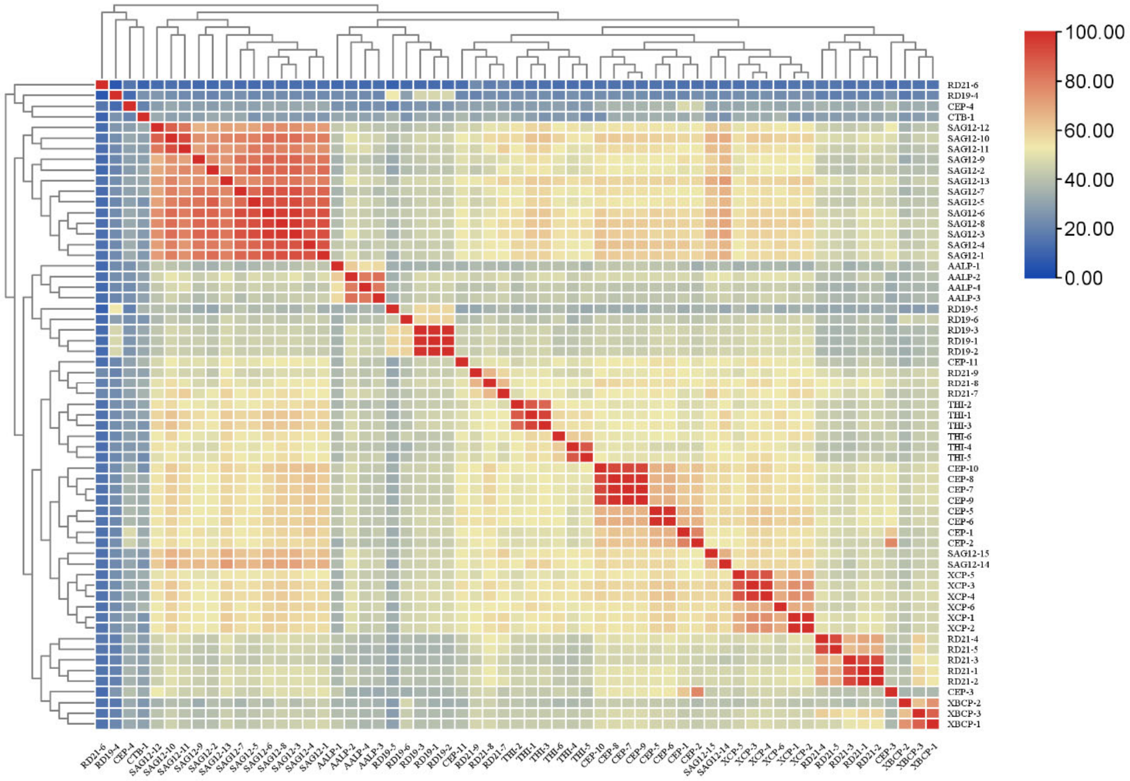

Figure S2. Pairwise protein sequence similarity matrix of ScPLCPs visualized using TBtools. Colors indicate percentage similarity, with the color scale bar shown in the upper right corner.

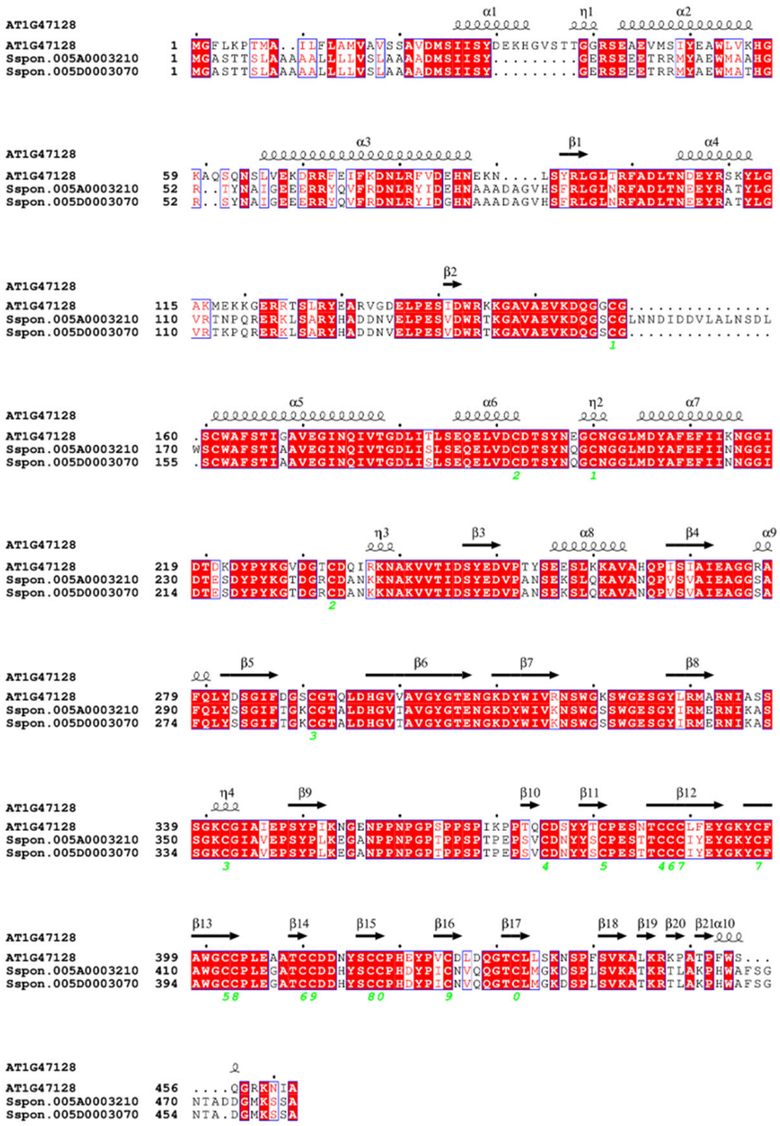

Figure S3. Multiple sequence alignment of SsRD21-4 (Sspon.005D0003070), SsRD21-5 (Sspon.005A0003210) and AtRD21A (AT1G47128) from sugarcane and Arabidopsis, generated with MEGA and visualized using ESPript 3.0. Strictly conserved residues are shaded red. Secondary structure elements are shown above the alignment based on the predicted three-dimensional structure of *Arabidopsis* AtRD21A. Disulfide bridges are indicated by green numbers below the sequences, with identical numbers denoting paired cysteine residues.
