# Supplementary material for: SsPit2A/B Effectors from Sporisorium scitamineum Interact with the Sugarcane PLCP ScRD21A and Reduce ScRD21A-Associated Cysteine Protease Activity via a Conserved LXRR Motif
Source: Plants (Basel). 2026 May 5;15(9):1408. doi: 10.3390/plants15091408 (PMC13165106; doi:10.3390/plants15091408)
Supplement: Supplementary file 1 [file plants-15-01408-s001.zip › Supplementary Materials/Supplementary Table .pdf]

Table S1. Primer sequences used in this study.

mScRD21A-VN-F	AGGTGGGTCAGGATCACTGCCGGAGTCCGTTGATTG
mScRD21A-VN-R	CGCGCCCCATGGATCCAGGGGGTACGATGGTTCAACG
SsPit2A(NSP)-VC-F	TCACGTGACGTCCGGATGATTCAGCTTCCCGCTATCCG
SsPit2A(NSP)-VC-R	CACCAGAACCTCCGGTATGAGAGCGACCCTGTTTCT
SsPit2B(NSP)-VC-F	AGAGGACACGCTCGAAGGCCTGTCATCGAAGGTCA
SsPit2B(NSP)-VC-R	AGCTCTTATACTCGAATACGTGCCTGGATTCGTTGG
GST-Pit2B(NSP)-F	CCCGGGTCGACTCGAGAGGCCTGTCATCGAAGGTCA
GST-Pit2B(NSP)-R	GATGCGGCCGCTCGATATACGTGCCTGGATTCGTTGG
GST-Pit2A(NSP)-F	CCCGGGTCGACTCGACATTCAGCTTCCCGCTATCCG
GST-Pit2A(NSP)-R	GATGCGGCCGCTCGAATGAGAGCGACCCTGTTTCT
AD-mScRD21A-F	GGAGGCCAGTGAATTGGAGTACCGCGCCACC
AD-mScRD21A-R	CGAGCTCGATGGATCCTGCTGCCCCATGAGTTCT
ScRD21A-F	ATGGGCGCCTCCACTACG
ScRD21A-R	TCATGCGCTGCTCTTCATGC
mScRD21A-F	CTGCCGGAGTCCGTTGATTG
mScRD21A-R	CAGGGGGTACGATGGTTCAA
ScRD21A-GFP-F	TCGACGACAAGACCGATGGGCGCCTCCACTACG
ScRD21A-GFP-R	TGAGGAGAAGAGCCGTGCGCTGCTCTTCATGCCA
BD-Pit2B-F	CATGGAGGCCGAATTCATGACAAACGCCCTCACGC
BD-Pit2B-R	GCAGGTCGACGGATCCTATACGTGCCTGGATTCGTTGGC
BD-Pit2A-F	CATGGAGGCCGAATTCATGCTGGTTCACTCAGCTCC
BD-Pit2A-R	GCAGGTCGACGGATCCTCAATGAGAGCGACCCTGTTTCT
Pit2B_mut-F	ACACATCAGCAGGTGCCGGAGGAACACCCGTCAA
Pit2B_mut-R	TTGACGGGTGTTCCTCCGGCACCTGCTGATGTGT
Pit2B(NSP)-mCherry-F	AGAACACGGGGGACGATGACAAACGCCCTCACGC
Pit2B(NSP)-mCherry-R	CTCACCAGGATCCCGGGCCCATACGTGCCTGGATTCGTTGG
Pit2A-mCherry-F	AGAACACGGGGGACGATGCTGGTTCACTCAGCTCC
Pit2A-mCherry-R	ACCATCAGGATCCCGGGCCCATGAGAGCGACCCTGTTTCT
Pit2B-mCherry-F	AGAACACGGGGGACGATGACAAACGCCCTCACGC
Pit2B-mCherry-R	CTCACCAGGATCCCGGGCCCATACGTGCCTGGATTCGTTGG
Pit2A(NSP)-mCherry-F	AGAACACGGGGGACGATGATTCAGCTTCCCGCTATCC
Pit2A(NSP)-mCherry-R	GATCCCGGGCCCGCGATGAGAGCGACCCTGTTTCT
Pit2A-F	ATGCTGGTTCACTCAGCTCC
Pit2A-R	ATGAGAGCGACCCTGTTTCT
Pit2A_mut-F	TGCAGCCAACGGCGCAGCTGGATGGTTTTGGAATT
Pit2A_mut-R	AATTCCAAAACCATCCAGCTGCGCCGTTGGCTGCA
Pit2B-F	ATGACAAACGCCCTCACGC
Pit2B-R	TATACGTGCCTGGATTCGTTGG
His-mScRD21A-F	AGGGATCCGAATTCGAGCTCCTGCCGGAGTCCGTT
His-mScRD21A-R	CCTGCAGGCGCGCCGCAGGGGGTACGATGGTTCAACGGCGATACT
qPCR-NbEF1a-F	ACGCACTGCTTGCTTTCA
qPCR-NbEF1a-R	AACCTCCTTCACGATTTCAT

qPCR-NbPTI5-F	CCTCCAAGTTTGAGCTCGGATAGT
qPCR-NbPTI5-R	CCAAGAAATTCTCCATGCACTCTGTC
qPCR-RBOHB-F	TTTTCTCTGAGGTTTGCCAGCCACCA
qPCR-RBOHB-R	GCCTTCATGTTGTTGACAATGTCTTT
qPCR-PAL-F	GTTATGCTCTTAGAACGTCGCCC
qPCR-PAL-R	CCGTGTAATGCCTTGTTTCTTGA
qPCR-NbAcre31-F	AATTCGGCCATCGTGATCTTGGTC
qPCR-NbAcre31-R	GAGAAACTGGGATTGCCTGAAGGA

Table S2. Representative HMMER command and filtering criteria used for PLCP retrieval

> hmmsearch.exe .-h
> hmmsearch.exe .\PF00112.hmm.\AP85_protein.fasta >out.txt

Table S3. MEME motif information associated with the eight conserved ScPLCP motifs.

**Motif**	**Sequence**
Motif1	DGTKYWIVKNSWGTSWGENGYIRMERGIS
Motif2	GCNGGLMDYAFEFIIKNGGJTTESDYPY
Motif3	AFSTVAAVEGINKIVTGKLVSLSEQEL
Motif4	DWREKGAVTPVKBQGQCGS
Motif5	CGTDLDHGVAAVGYG
Motif6	PANNEAALQKAVANQPVSVAIDAGG
Motif7	LNQFADLTNEEFRATYLGKR
Motif8	YGRVYKDAGEKARRFEVFKDNVRFIESHN

Table S4. Mutation sites and amino acid changes introduced in SsPit2A and SsPit2B.

	SsPit2A	SsPit2B
Original	……AANLERRWFW……	……DTSLDRRGTP……
Mutant	……AANGAAGWFW……	……DTSAGAGGTP……
